# A Survey of Common Payment Methods and Their Determinants in Dental Clinics, in Tehran, 2018

**DOI:** 10.1055/s-0039-1697556

**Published:** 2019-12-31

**Authors:** Reza Emrani, Katayoun Sargeran, Justein Grytten, Hossein Hessari

**Affiliations:** 1 Department of Community Oral Health, School of Dentistry, Tehran University of Medical Sciences, Tehran, Iran; 2 Research Center for Caries Prevention, Dentistry Research Institute and Department of Community Oral Health, School of Dentistry, Tehran University of Medical Sciences, Tehran, Iran; 3 Institute of Community Dentistry, University of Oslo, Oslo, Norway

**Keywords:** fixed salary, fee-for-service, payment method, dentistry

## Abstract

**Objective** It is believed that the payment method to dentists may affect their treatment decisions. Although payment systems may enhance job satisfaction, reduce the costs, and make better treatment decisions, there is little information about how to achieve these objectives. The aim of the present study was to survey the payment methods and the related factors in dental clinics of Tehran.

**Materials and Methods** Using the latest list published by the Iranian Ministry of Health, we visited all dental clinics located in Tehran, and used a checklist to collect the data of the type of management, geographical location of the clinic in Tehran, payment method and its amount, history of payment method changes in the last decade, relationship between the amount of payment and the position of technical supervisor dentist, any difference in the amount of payment to male and female dentists and to young and experienced dentists. Then, the relationship between the amount of payment and the above-mentioned variables was investigated.

**Results** The governmental sector tends to use fixed salary methods and the private sector usually uses the fee-for-service (FFS) method. Geographical location, type of management, date of establishment, and having the position of technical supervisor dentist had a significant relationship with the amount of payment. The dentist’s gender and years of experience did not have any relationship with the amount of payment.

**Conclusion** According to the results of the present study, the method and amount of payment to dentists were related to the date of clinic establishment, having the position of technical supervisor dentist, and geographical location of the clinic. These factors could be considered as the main elements in balancing costs in clinics and improving job satisfaction among dentists.

## Introduction

Economic theories and common sense, both suggest that the way people are paid affects their working patterns. 1 Dental services are one of the health services that have undergone less economic analysis. Therefore, proper economic analysis of dental services may reduce their costs and provide a good solution for improving their efficiency. One of the most important elements in providing dental services is the method of payment to dentists. 2 3 In recent years, it has been observed that changes in the payment method and rewards affect the distribution of services and the level of dentists’ satisfaction and quality of service. Attention should be paid to payment methods when health systems focus on balancing their costs and reducing unnecessary costs. 4 Today, in many countries, public health, including dental care, faces many economic problems. For this reason, any small disorder in the health economics may lead to serious problems in the service delivery system. 5

Since, doctors’ salary is one of the costliest payments in the health system, it is necessary to enhance the cost-effectiveness of these payments due to the limited resources of the health sector. The effect of healthcare professionals’ income on maintaining and improving the efficiency, accessibility, and quality of service delivery is one of the health system concerns. 6

Payment methods play a major role in the overall health and governments have different responsibilities in this regard. Governments have become fully aware of the importance of economic incentives and have made adjustments to payment methods in many ways. Moreover, they use economic rewards to increase the productivity of the dental health sector. For example, studies have shown improvements in caries prevention programs after changing the payment method, which indicates the importance of the economic incentives and the role of the payment method in upgrading the system. 7

The main payment models for dental care providers include (1) fixed payments for specified services called “salary” and (2) payment systems based on the number of specified services, called “per-case” or “fee-for-service (FFS),” which is common in the world. 8 Moreover, to improve the system performance, a combination of these two methods is sometimes applied known as mixed payment. Different countries have different payment methods, and usually change these methods at different time intervals. The fixed salary system is associated with a lower workload and an unreasonable increase in the duration of treatment, more referral, and increased absenteeism in the workplace. However, this system decreases the organization’s costs. The FFS method of payment is associated with a higher workload and unnecessary treatments and visits; however, it is more precise and encourages the presence of the doctor in the workplace. It should be noted that an ideal payment system has not yet been developed. The issue of frequent referral in the fixed salary system and higher risk acceptance are also indicators of the FFS payment system. 9

Increasing unnecessary treatments or reducing the required treatments and excessive referrals over the payment method can lead to increased treatment costs for the patient and pressure on public and private insurance. Choosing the correct payment method can reduce unnecessary referrals and possible nonreferral and increase health cost-effectiveness. 10

Investigating the relationship between the performance of the dentists and the payment method can help design proper health care plans. In Iran, a large part of the health sector’s expenditures is related to human resources, especially physicians. The methods and mechanisms of paying the physician fee can have a significant effect on the health costs of the country and providing high-quality medical care. In Iran, FFS and fixed salary are the most common payment systems. Of course, in some governmental health care centers, remuneration and bonus are also used to motivate the provider. A mixed method is used in the Organization of Armed Forces of Iran and some other private centers. 11

Dental clinics in Iran provide dental services to patients, separately; some other dental centers offer dental services together with medical services in a general medical clinic. The dental clinics must be at least 150 m ^2^ in area and have five units and three general dentists.

Surveying the characteristics of dental clinics and their payment method must be considered as one of the foundational elements in the dental cost analysis. Therefore, due to lack of payment method studies in Iran and lack of information on payment methods in Tehran clinics, we conducted a study to examine the status of the payment method in dental clinics of Tehran. The aim of the present study was to survey the payment method and the related factors in dental clinics of Tehran.

## Materials and Methods

In this cross-sectional study, the latest list of Tehran dental clinics published by the Iranian Ministry of Health was used to visit all dental centers in Tehran, commonly known as dental clinics. Contact was made via telephone, if it was difficult to visit the clinic or if the managers were absent. Dental services are either provided in a dental department in medical clinics or in clinics that only offer dental services. In the present study, we selected those clinics that only offered dental services. Additionally, dental clinics should have at least three dentists, five dental units, a single fund, and a mechanism to pay the dentists.

This study was approved by the Ethics Committee of School of Dentistry, Tehran University of Medical Sciences (code of ethics 42794260). The heads of the dental clinics agreed to participate in the research.

To achieve the objectives of the study, a checklist was used to collect the data of the physical characteristics of the building, number of working dentists and their background, geographical location of the clinic in Tehran, type of contract with dentists (written contract, oral contract), payment method and its amount, and possible changes of the payment method in the last decade. The checklist was also used to assess the amount of payment and the technical responsibilities of the dentists and their specialty, contract between insurance companies and clinics, number of dental nurses, possible difference in payment to male and female dentists and also to young and experienced dentists, number of administrative staff, number of dental units, presence of oral radiology section, and presence of a panoramic radiology section.

Questions were asked verbally from the clinic directors and the responses were immediately recorded in the checklist. The data of 67 dental clinics of Tehran and their payment methods were analyzed in the present study.

Dental clinics must have a technical supervisor in every working shift. This person is responsible for all treatment procedures and supervises other dentists and nurses. Dental clinics usually have to work 24 hours and offer general and specialized dental services. Charity clinics were not included in this study because the dentists in these clinics usually provide subsidized dental care. The geographical distribution of registered dental clinics was set according to the development index. 12 Chi-square test was applied for data analysis using SPSS version 21.

## Results

The data of 56 out of 67 registered dental clinics were recorded (response rate = 83%). The majority (79%) of the dental clinics in Tehran were owned by the private sector. The payment method was FFS in almost all private dental clinics (93%). The current payment system was fixed salary in most of the public dental clinics ( Table 1 ).

**Table 1:** Ownership status of registered dental clinics n (%) according to payment method in Tehran, Iran, 2018

Ownership status	Salary	FFS	Mixed	History of change	Total
Governmental	4 (7)	3 (5)	3 (5)	3	10
Private	1 (1.5)	40 (71)	3 (5)	29	44
Semi-private	0	1 (1.5)	1 (1.5)	0	2
Total	5 (8.5)	44 (78)	7 (12.5)	32	56
Abbreviation: FFS, free for service.						

There was a significant relationship ( *p* = 0.001) between the management type and payment method. The most common payment method in the governmental and private sector was salary and FFS, respectively. Changes in the payment method were seen in 32 dental clinics. According to the geographical location of dental clinics, the majority of dental clinics were located in the north (more affluent part; 39.3%), followed by the center (semi-affluent part; 28.4%), and the west (semi-affluent part) of Tehran (21.3%; Table 2 ).

**Table 2:** Geographical distribution of registered dental clinics (n = 56) according to payment methods in Tehran, Iran, 2018

	Salary		FFS		Mixed		Total	
	*n*	%	*n*	%	*n*	%	*n*	%
North (affluent)	1	1.78	21	37.5	0	0	22	39.3
West (semi-affluent)	0	0	2	3.5	0	0	2	3.5
Centre (middle)	4	7.1	9	16	3	5.3	16	28.4
East (lower middle)	0	0	10	17.8	2	3.5	12	21.3
South (poor)	0	0	1	1.78	1	1.78	2	3.5
Suburbs (very poor)	0	0	1	1.78	1	1.78	2	3.5
Total	5	8.9	44	78.5	7	12.5	56	100
Abbreviation: FFS, free for service.								

Table 3 shows the distribution of the geographical location of dental clinics and their payment method. There was a significant relationship ( *p* = 0.04) between the location of the clinics and the amount of payment, and dental clinics located in more affluent areas paid lower salaries ( Table 3 ).

**Table 3:** Geographical distribution (n = 56) and amount of payment to the dentists (share percentage of a dentist) in dental clinics of Tehran, Iran, 2018

	Share percentage of a dentist						
	40%	42%	44%	45%	50%	55%	60%
North (affluent)	7	4	2	8	0	0	0
West (semi-affluent)	0	0	0	0	2	0	0
Centre (middle)	1	1	0	4	5	1	0
East (lower middle)	2	0	0	4	6	0	0
South (poor)	0	0	0	1	1	0	0
Suburbs (very poor)	0	0	0	0	0	0	2
Total	10	5	2	17	14	1	2
Note: Statistical analysis by Chi-square test, *p* = 0.04, df = 30, χ ^2^ = 78.02.							

The results showed no relationship ( *p* = 0.573) between the amount of payment and work experience. There was a significant relationship between having the position of technical supervisor dentist and the amount of payment. A technical supervisor dentist had higher income than other dentists who did not have any technical responsibilities in the clinic. There was no difference in payment to male and female dentists in payment system and 52 clinics paid male and female dentists equally ( Table 4 ).

**Table 4:** Relationship between amount of payment gender, work experience, and technical manager, in Tehran, Iran, 2018

			Share percentage of a dentist (Ordinal variables ranged from 40 to 60%)		
		*n*	df	χ ^2^	*p* -Value
Working experience	Yes	39	6	3.52	0.57
	No	17			
Having technical manager job	Yes	47	6	12.68	0.04
	No	9			
Gender inequality in payment	Yes	4	1	8.91	0.01
	No	52			
Abbreviation: df, degree of freedom. Note: Statistical analysis by Chi-square test. Share percentage was divided in three groups, (40–44, 45–50, and 55–60%).					

There was a significant relationship between the establishment date of the clinic and the amount of payment and long-established clinics paid less than new clinics.

## Discussion

This study was conducted to evaluate the current payment methods and the related factors in dental clinics of Tehran. The results of the study showed that governmental systems tend to use a fixed salary payment method and private systems tend to use the FFS system. In many countries, the government has a little share of dental services. 13

Our results also showed that the majority of Tehran dental clinics were private and the government had a little role in this section.

The FFS payment system is popular among private systems because with FFS financing, the dentist is remunerated according to the actual cost of the treatment; therefore, it is easier to ensure quality with this system than with other systems. 14 15 It should be considered that private clinics need to balance their income and costs, and their survival depends on their income because they do not have any governmental support. The sum of these factors makes private systems choose the FFS as the payment method.

The result of our study showed that 32 clinics had a history of changing the payment method in their records. This is explained by the fact that work and economic conditions are changing, so clinics, in different situations, try to make changes to achieve better conditions. At the macro level, it is also frequently observed that governments and health policymakers have changed the payment method to improve the quality of health services. 16

Our study showed that longer-established clinics usually paid lower salaries to dentists. This can be explained by the fact that these centers, which had a higher turnover of patients, were very popular among dentists and many of them were willing to work in these centers because these clinics can control the financial conditions to their advantage.

Clinics located in the more affluent areas of the city paid a lower percentage to dentists. On the other hand, dental services fees are higher in these regions, indicating that a dentist usually works less hours compared with dentists in other regions, but earns a similar or higher income. It may be considered as one of the most important factors why dentists tend to work in these clinics.

According to the results of this study, technical supervisor dentists received higher salaries. Since, technical supervisor dentists are responsible for the whole shift and must be present in the clinic at all times, their salary is higher than other dentists.

There was no difference in payment to male and female dentists, indicating that gender was not an effective factor in payment in Tehran dental clinics.

The number and proportion of women entering the profession of dentistry have increased during the past four decades, as evidenced by registrations with the General Dental Council. The proportion of registrations by women increased from 25% of new entrants in 1975 to over one third in 1985 to one half in 1991 in Britain. 17 Some studies have examined differences between male and female dental practitioners in their work patterns. Our finding showed that male and female dentists received similar percentage in Tehran dental clinics in FFS system but in the public sector, the female dentists salaries were less than men, but in the private sector, the share percentage of both were equal, although the total income of men was higher.

The work experience of the dentists did not correlate with their payments. Clinics paid young and experienced dentists similarly. It should be considered that experienced dentists mainly prefer working in their own private clinics, while, younger graduates work in public and private clinics, so more work experience may not be an effective factor.

A limitation of our study was lack of sufficient information about the income of dentists. The only information we had was the percentage they received, and not the exact amount of payment. Obviously, it is not appropriate to ask about income, but it can be assumed that the amount of payment may also be effective in job satisfaction and providing high-quality services. This matter is important for both clinics and dentists.

A strong point of our study was that it was done in dental centers, where the managers were also dentists and there were no other sections other than dental clinics, so only factors related to this profession were analyzed.

The results of our study were similar to the those of a study in the United Kingdom that evaluated factors affecting the working patterns of orthodontists. The authors found that the distribution of the orthodontists in the United Kingdom was not equitable and some regions suffered from an unfavorable ratio of orthodontists. Moreover, this geographical distribution was an effective factor in the income of dentists. 18

The result of another study in the USA showed that dentists who worked in remote regions like Alaska had more income and geographical location was reported to be an effective factor in dentistry. 19

Although the payment method has an important impact on the dentist’s treatment decisions and many factors affect the method and amount of payment to dentists in clinics, it should be noted that development of moral standards could be considered an appropriate strategy for controlling this effect. It is important to establish a culture among dentists, policymakers, and managers of dental clinics to focus on the ethical aspects of service provision. Norms should be established within the organization that counteracts any tendency for dentists to be tempted to achieve financial gains at the expense of the patient. 20

The results of this study can be a good guide for healthcare policymakers in the field of dentistry. This study showed some relationships between employers and dentists and it is necessary to know how to make the next modification.

Similar studies seem to be necessary every few years because oral health policies are changing, and these changes may affect the dentists’ salaries. Furthermore, the type of payment systems may also change. For example, in several countries, per capita payments and pay-for-performance payments have been introduced. These are meant to improve quality and effectiveness of dental services. 21 Such schemes have not yet been introduced in Teheran. However, they may be implemented in the future. In that case, surveys should be performed so that the effectiveness of these new payment systems can be assessed.

## Conclusion

This study found that both governmental and private systems consider many factors to design an appropriate payment system. Patient load and location of the clinic affect the managers’ decision regarding the amount of payment to dentists. Each method has some advantages and disadvantages. Clinics (especially private ones) tend to increase their income and take into account several factors to design a good payment system. We believe more studies are required to elaborate the factors affecting the payment system.
